# Association between primary care appointment lengths and subsequent ambulatory reassessment, emergency department care, and hospitalization: a cohort study

**DOI:** 10.1186/s12875-022-01644-8

**Published:** 2022-03-06

**Authors:** Kristi M. Swanson, John C. Matulis, Rozalina G. McCoy

**Affiliations:** 1 Robert D. and Patricia E. Kern Center for the Science of Health Care Delivery, Mayo Clinic, Rochester, USA; 2 Division of Community Internal Medicine, Department of Medicine, Mayo Clinic, Rochester, USA; 3 Division of Health Care Delivery Research, Robert D. and Patricia E. Kern Center for the Science of Health Care Delivery, Mayo Clinic, Rochester, USA

**Keywords:** Schedules and Appointments, Primary Care, Practice Patterns, Healthcare Utilization, Quality of Healthcare

## Abstract

**Background:**

To meet increasing demand, healthcare systems may leverage shorter appointment lengths to compensate for a limited supply of primary care providers (PCPs). Limiting the time spent with patients when evaluating acute health needs may adversely affect quality of care and increase subsequent healthcare utilization; however, the impact of brief duration appointments on healthcare utilization in the United States has not been examined. This study aimed to assess for potential inferiority of shorter (15-min) primary care appointments compare to longer (≥ 30-min appointments) with respect to downstream healthcare utilization within 7 days of the initial appointment.

**Methods:**

We performed a retrospective cohort study using electronic health record (EHR), billing, and administrative scheduling data from five primary care practices in Midwest United States. Adult patients seen for acute Evaluation & Management visits between 10/1/2015 and 9/30/2017 were included. Patients scheduled for 15-min appointments were propensity score matched to those scheduled for ≥ 30-min. Multivariate regression models examined the effects of appointment length on repeat primary care visits, emergency department (ED) visits, hospitalizations, and diagnostic services within 7 days following the visit. Models were adjusted for baseline patient, visit, and provider characteristics. A non-inferiority approach was employed.

**Results:**

We identified 173,758 total index visits (6.5% 15-min, 93.5% ≥ 30-min). 11,222 15-min appointments were matched to a comparable ≥ 30-min visit. Longer appointments were more frequent among trainee physicians, patients with limited English proficiency, and patients with more comorbidities. There was no significant effect of scheduled appointment length on the incidence of repeat primary care visits (OR = 0.983, CI: 0.873, 1.106) or ED visits (OR = 0.856, CI: 0.700, 1.047). Shorter appointments were associated with lower rates of subsequent hospitalizations (OR = 0.689, CI: 0.504, 0.941), laboratory services (OR = 0.682, CI: 0.643, 0.724), and diagnostic imaging services (OR = 0.499, CI: 0.466, 0.534). None of the non-inferiority thresholds were exceeded.

**Conclusions:**

For select indications and select low risk patients, shorter duration appointments may be a non-inferior option for scheduling of patient care that will not result in greater downstream healthcare utilization. These findings can help inform healthcare delivery models and triage processes as health systems and payers re-examine how to best deliver care to growing patient populations.

**Supplementary Information:**

The online version contains supplementary material available at 10.1186/s12875-022-01644-8.

## Background

A robust primary care infrastructure is foundational for individual and population health [1]. The growing shortage of primary care providers (PCPs) impedes timely and effective management of acute and chronic health conditions, delivery of essential preventive services, and careful stewardship of healthcare resources [1, 2]. It also threatens future progress in caring for an aging population [3–5]. This shortage is driven, in part, by increasing demands for PCP services, fueled by population growth, increasing prevalence of chronic health conditions, and falling rates of the uninsured [6–8]. Furthermore, alternative payment models place greater responsibility on primary care practices to complete work outside the traditional purview of primary care, including population health management and care coordination [9, 10].

To improve healthcare access for growing patient populations and maximize revenue generation in a fee-for-service environment, many healthcare systems have introduced shorter appointment lengths (e.g., 15-min in duration) into scheduling templates, seeking to maximize the number of patients seen on a given day [11, 12]. While shorter appointment lengths do allow more patients to be seen in a given day, allocating valuable clinician time in standardized, brief increments may not effectively meet patient needs, resulting in incomplete or incorrect diagnostic evaluations, poor patient experience, and potentially avoidable downstream healthcare utilization [13–20]. Despite scheduled appointment lengths getting shorter, the time required to care for increasingly complex patients and comply with growing regulatory and documentation requirements has been increasing. A National Ambulatory medical survey suggested that physician reported time spent directly with patients had lengthened by an average of 2.4 min between 2008 and 2015, raising the question of what amount of time is needed for clinicians to provide satisfactory care [12, 21].

Although there is no consensus on what constitutes “adequate time” with a clinician, shorter visits may be inadequate to effectively address patient concerns and also manage chronic health conditions, deliver necessary preventive services, and interact with the electronic health record (EHR) [13, 14, 22–27]. Prior work in evaluating the effects of appointment length on healthcare utilization is sparse, often conflicting, and dated [28–33]. When shorter duration visits are employed, the quality of care rendered is uncertain and rarely rigorously evaluated in the primary care setting. Wilson et al. performed two separate systematic reviews, noting that shorter appointment lengths were associated with missing patient care elements among British General Practitioners [29, 32]. The impact of shorter duration primary care office visits on subsequent healthcare utilization in the United States has not been examined.

A more nuanced assessment of the value of different duration appointment lengths in primary care is needed as health systems and payers re-examine how to best deliver care to complex patient populations. Using data from an integrated healthcare delivery system in the Midwestern United States, we aimed to assess for potential inferiority of short (15-min) appointment lengths in the primary care setting compared to longer (≥ 30-min or greater) appointments by examining downstream healthcare utilization including return office visits, emergency department and hospital utilization and diagnostic testing in the 7 days following the initial appointment. Results of this study will help inform healthcare delivery models and the appropriateness of using shorter appointment slots in the primary care setting.

## Methods

### Study design & setting

We performed a retrospective cohort study using EHR, billing, and administrative scheduling data from Mayo Clinic, Rochester, Minnesota, U.S.A. Mayo Clinic is an integrated healthcare delivery system that serves local, regional, national, and international patients. The five primary care practices of Mayo Clinic Rochester reside in both urban and rural areas and are comprised of family medicine, internal medicine, and pediatrics specialties that care for over 150,000 local residents, Mayo Clinic employees, and their dependents. PCPs in these practices include attending physicians, trainees in medical education programs (residents and fellows), nurse practitioners (NPs), and physician assistants (PAs). This study was approved by Mayo Clinic’s Institutional Review Board.

### Study population

We identified acute outpatient office (“index”) visits among adults (age ≥ 18 years) in the community internal medicine (CIM) and family medicine (FM) practices of Mayo Clinic, Rochester between 10/1/2015 and 9/30/2017. Patients were required to be empaneled to a Mayo Clinic PCP for at least one year prior to the index visit and for 30 days following (to allow for ascertainment of baseline characteristics and utilization outcomes). Index visits were first identified using Current Procedural Terminology (CPT) codes for office or other outpatient evaluation and management (E&M) visits (99201–99215, 99241–99245) and did not include preventive medicine, case or care management services, special evaluations, advanced care planning, or services performed outside the office setting. Index visits were then merged with administrative scheduling data to determine the allotted appointment time.

To ensure visits were for a new chief complaint, we excluded visits that were preceded by another eligible E&M visit in primary care within the previous two weeks. Visits for preventive services only or with non-PCP providers (e.g., nurse, dietician, social worker, etc.) were excluded from analyses, as were visits where the scheduled appointment length could not be determined. Patients who did not provide research authorization were excluded in accordance with Minnesota state law [34].

### Explanatory variable

The exposure of interest was the scheduled appointment length of the index visit. Appointment lengths were ascertained from administrative scheduling data and categorized as 15-min versus ≥ 30-min. Appointment lengths are determined by centralized scheduling staff members using standardized templates based on the patient’s stated health concern and patient characteristics (see Additional file 1); the vast majority of appointments are either 15 or 30 min, but 45-min appointments are available for patients new to the practice and those requiring interpreter services. Because only 2.3% of longer appointments were 45 min long, they were grouped together with the 30-min appointments. Schedulers may substitute their own judgement and schedule 15-min concerns into longer appointment slots. Additionally, providers can request that concerns normally scheduled in a longer appointment be placed into a 15-min slot based on their calendar availability. As such, there is substantial overlap of clinical conditions and contexts that may be seen in either 15-min or 30-min time slots.

### Independent variables

Covariates of interest included patient, visit, and provider level characteristics. Patient characteristics were extracted from the EHR and included age, ethnicity, race, gender, marital status, and geographic location. Marital status was included as a proxy for social support. Limited English proficiency was identified using the language preference recorded in the patient’s registration data. The Deyo adaptation of the Charlson comorbidity index with incorporated severity weighting was calculated using ICD-9/ICD-10 diagnosis codes from billing data [35–37]. Prior healthcare utilization, measured by the number of emergency department (ED) visits and hospitalizations in the prior year, was also obtained for each index visit.

Visit and provider information included the specialty area of the appointment (FM vs. CIM), the type of provider seen (physician, NP/PA, or trainee physician), and the specific clinic site where care was sought. The chief complaint for the visit was obtained using the primary diagnosis from billing data and was summarized using the clinical classification software refined (CCSR) multi-level categories [38].

### Outcomes

Outcomes of interest were assessed within 7 days following the index visit and included outpatient office visits in primary care (referred to as repeat visits), ED visits, hospitalizations, laboratory services, and diagnostic imaging services. Repeat visits were identified using similar methodology to that of the index visit, but without examination of the two weeks prior. ED visits were identified using CPT codes (99281–99288). Laboratory and diagnostic imaging services were identified using revenue center codes (i.e., codes used to identify accommodation or ancillary services), and in some instances, a combination of revenue center and CPT codes (see Additional file 2). To account for the fact that diagnostic services may, in some instances, be ordered and conducted ahead of the scheduled appointment, we performed a sensitivity analysis excluding laboratory or imaging services rendered on the same day as the index appointment.

### Adjustment for differences between groups

We anticipated that certain factors would be influential in determining whether a patient gets scheduled for a 15-min versus ≥ 30-min appointment. These would include patient level factors (medical complexity, social support, utilization patterns) and system factors (triage factors, access to care). For this reason, we implemented propensity score matching to account for potential selection bias in the exposure of interest. Full details regarding the propensity score matching approach used are provided in Additional file 3.

### Statistical analysis

Patient, visit, and provider characteristics were compared using standardized differences as opposed to p-values, as examination of standardized differences is a more appropriate method for determining balance across matched groups that is not influenced by reductions in sample size due to matching [39]. Crude outcome rates were compared within our matched population using McNemar’s test for paired data.

Multivariate regression models were used to examine the effects of appointment length on each of the outcomes of interest, while adjusting for important confounding variables not used as part of the matching process. We used conditional logistic regression methods to account for the matched nature of the data. Confounding factors included the ethnicity, race, gender, and marital status of the patient, as well as prior healthcare utilization. We reported multivariate regression results in the form of odds ratios and 95% confidence intervals.

To assess non-inferiority of shorter appointment lengths, we a priori defined a non-inferiority threshold of 10% increased likelihood (Odds Ratio [OR] of 1.1) of subsequent ED and hospital visits and a threshold of 20% (OR of 1.2) increased likelihood of repeat visits, laboratory services, and diagnostic imaging services. In essence, we are willing to accept a higher likelihood of subsequent utilization for those with shorter appointments so long as the increased rate does not exceed our defined non-inferiority threshold. We used the upper limits of the 95% confidence intervals as a boundary for assessing non-inferiority and compared this value to the corresponding non-inferiority threshold. All data management and analyses were carried out using SAS 9.4 (SAS Institute Inc. Cary, NC).

## Results

We identified 173,758 eligible acute care visits to primary care during the study period. Shorter (15-min) appointments accounted for 6.5% (*N* = 11,222) of the visits, while appointments scheduled for ≥ 30-min comprised 93.5% (*N* = 162,536). Prior to matching, the majority (63.8%) of 15-min appointments were scheduled in the FM practice, while longer appointments were more evenly distributed between the practice areas (52.1% in FM and 47.9% in CIM) (Table 1). Longer appointments were more frequently scheduled with trainee physicians and for patients with limited English proficiency and a higher number of comorbidities. Visits with chief complaints related to congenital anomalies, mental illness, blood diseases, the circulatory system, digestive system, or musculoskeletal system, as well as endocrine or metabolic diseases, immunity disorders, injuries, and ill-defined conditions were more likely to have a longer appointment scheduled.

**Table 1:** Distribution of Population Characteristics and Propensity matched covariate balance

Characteristic	Full Population				Matched Cohort			
	**Total** ***N* = 173,758**	**15 Minutes ** ***N* = 11,222**	** ≥ 30 Minutes** ***N* = 162,536**	**Standardized Difference**	**Total** ***N* = 22,444**	**15 Minutes ** ***N* = 11,222**	** ≥ 30 Minutes** ***N*** **= 11,222**	**Standardized Difference**
**Age (mean; SD)**	54.2 (19.3)	51 (31)	55 (31)	20.6	50.4 (19.2)	51 (31)	50 (31)	1.6
**Practice Area (%)**								
Family Med	52.9	63.8	52.1	23.7	64.0	63.8	64.2	0.9
PCIM	47.1	36.2	47.9	23.7	36.0	36.2	35.8	0.9
**Provider Type (%)**								
Physician	54.7	60.6	54.3	12.9	60.7	60.6	60.8	0.3
NP/PA	29.5	31.3	29.4	4.2	31.1	31.3	30.9	1.1
Resident	15.8	8.0	16.3	25.5	8.2	8.0	8.3	1.2
**Patient Language (%)**								
English	96.1	98.6	95.9	18.9	98.5	98.6	98.4	2.4
Non-English	3.7	1.1	3.9	20.2	1.1	1.1	1.1	1.8
Unknown	0.2	0.3	0.2	1.7	0.4	0.3	0.5	1.8
**Clinic Site (%)**								
Clinic A	54.8	42.0	55.7	27.7	42.6	42.0	43.1	2.4
Clinic B	10.7	14.2	10.5	11.2	14.2	14.2	14.1	0.2
Clinic C	16.2	19.6	16.0	9.4	19.8	19.6	20.0	1.0
Clinic D	10.2	13.4	10.0	10.7	13.3	13.4	13.1	0.9
Clinic E	8.0	10.8	7.8	10.3	10.2	10.8	9.6	4.1
**Charlson Index (%)**								
** 0**	49.5	60.3	48.8	23.3	61.4	60.3	62.5	4.5
** 1**	19.2	18.0	19.3	3.5	17.9	18.0	17.7	0.6
** 2**	9.6	8.0	9.7	5.9	7.7	8.0	7.3	2.8
** 3**	6.7	4.8	6.8	8.8	4.5	4.8	4.1	2.9
** 4**	4.2	2.5	4.3	9.7	2.4	2.5	2.3	1.7
** 5 or more**	10.8	6.4	11.1	16.6	6.3	6.4	6.1	1.4
**Disease Indication (%)**								
Complications of Pregnancy; childbirth; and the puerperium	1.2	1.8	1.2	4.6	1.8	1.8	1.8	0.3
Congenital Anomalies	0.1	0.02	0.09	3.0	0.0	0.02	0.0	1.9
Diseases of the blood and blood-forming organs	0.4	0.3	0.4	2.3	0.3	0.3	0.2	1.7
Diseases of the circulatory system	8.6	2.8	9.0	26.4	2.9	2.8	2.9	0.2
Diseases of the digestive system	5.0	3.2	5.1	9.3	3.3	3.2	3.3	0.3
Disease of the genitourinary system	6.6	7.0	6.6	1.6	7.0	7.0	6.9	0.5
Diseases of the musculoskeletal system and connective tissue	18.6	4.3	19.6	48.5	4.4	4.3	4.4	0.5
Diseases of the nervous system and sense organs	8.3	14.8	7.9	22.1	14.9	14.8	14.9	0.3
Diseases of the respiratory system	11.2	23.6	10.3	35.9	24.0	23.6	24.4	1.7
Diseases of the skin and subcutaneous tissue	6.1	18.7	5.2	42.7	18.7	18.7	18.7	0.1
Endocrine; nutritional; and metabolic diseases and immunity disorders	7.3	1.9	7.7	27.3	2.0	1.9	2.0	0.2
Infectious and parasitic diseases	2.1	6.1	1.8	22.1	5.9	6.1	5.7	1.7
Injury and poisoning	4.0	3.4	4.0	3.3	3.5	3.4	3.5	0.5
Mental Illness	7.6	1.7	8.0	29.9	1.7	1.7	1.7	0.8
Neoplasms	1.2	2.5	1.1	11.1	2.3	2.5	2.0	3.4
Residual Codes; unclassified	1.8	0.5	1.9	12.9	0.5	0.5	0.4	2.3
Symptoms; signs; and ill-defined conditions and factors influencing health status	9.9	7.3	10.1	9.7	7.4	7.3	7.4	0.1
**Additional characteristics not used in the matching process**								
** Ethnicity (%)**								
Hispanic	1.9	1.7	2.0	2.1	1.7	1.7	1.7	0
Not Hispanic	95.3	95.3	95.3	0.1	95.3	95.3	95.3	0.3
Unknown	2.8	3.0	2.8	1.6	3.0	3.0	3.0	0.4
** Race (%)**								
African American	3.3	1.5	3.4	12.8	1.7	1.5	1.9	3.3
Asian	2.8	2.3	2.9	3.4	2.4	2.3	2.4	0.6
Other/Unknown	4.0	3.5	4.0	3.0	3.3	3.4	3.1	1.8
White	89.9	92.8	89.7	10.9	92.7	92.8	92.6	0.8
** Sex (%)**								
Female	61.9	62.7	61.8	1.9	62.6	62.7	62.5	0.6
Male	38.1	37.3	38.2	1.9	37.4	37.3	37.5	0.6
** Marital Status (%)**								
Married	62.3	65.1	62.2	6.1	63.3	65.1	61.6	7.3
Not Married or Unknown	37.7	34.9	37.8	6.0	36.6	34.9	38.4	7.2
** Geographic Location (%)**								
Olmsted County	75.0	72.0	75.2	7.4	72.6	72.0	73.2	2.8
SE Minnesota	23.4	26.7	23.2	8.1	26.0	26.7	25.2	3.3
Outside SE MN	1.6	1.4	1.6	2.1	1.5	1.4	1.6	1.8

We matched 11,222 15-min appointments to a comparable ≥ 30-min appointment visit, resulting in a final matched cohort of *N* = 22,444 visits. After performing one-to-one propensity score matching, substantial balance was achieved between the two groups, with all match characteristics having a standardized difference below 5% (Table 1). Differences in additional baseline demographic characteristics not used as part of the matching process also substantially improved after matching.

There were no significant differences in the crude rates of repeat acute care visits between the appointment length groups (Table 2). Shorter appointment lengths had a lower rate of 7-day ED visits (1.8% vs. 2.2%, *p* = 0.03) and hospitalizations (0.7% vs. 1.0%, *p* < 0.01) compared to longer appointment lengths. Longer appointments were also followed by higher rates of laboratory (38.3% vs. 30.5%, *p* < 0.001) and diagnostic imaging services (28.2% vs. 17.2%, *p* < 0.001). These findings held true when excluding same day diagnostic services as part of our sensitivity analyses (see Additional file 4, Tables D5 and D7).

**Table 2:** Summary of crude outcome rates (within 7 days) across appointment length

Characteristic	Appointment Length		*P*-Value
	**15 Minutes** ***N* = 11,222**	**30 Minutes** ***N* = 11,222**	
	**N (%)**	**N (%)**	
**Repeat Visits**	605 (5.39)	619 (5.52)	0.6807
**ED Visits**	199 (1.77)	243 (2.17)	0.0345
**Hospitalizations**	78 (0.70)	116 (1.03)	0.0061
**Diagnostic Laboratory**	3,424 (30.51)	4,299 (38.31)	< 0.0001
**Diagnostic Laboratory (not including same day)**	1,418 (12.64)	1,664 (14.83)	< 0.0001
**Diagnostic Imaging**	1,928 (17.18)	3,161 (28.17)	< 0.0001
**Diagnostic Imaging (not including same day)**	1,092 (9.73)	1,769 (15.76)	< 0.0001

**Fig. 1:**
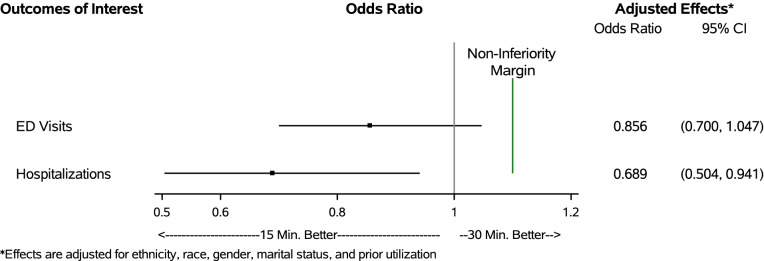
Effect of primary care appointment length on ED visits and hospitalizations within 7 days of the index appointment

**Fig. 2:**
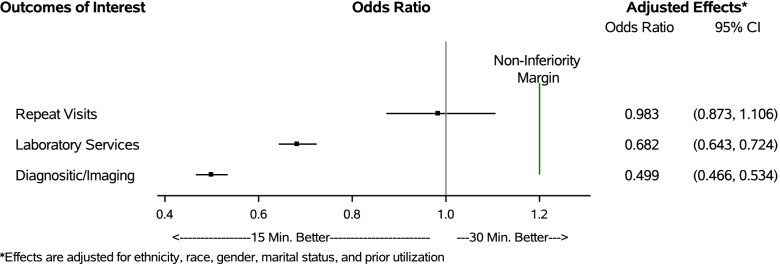
Effect of primary care appointment length on repeat visits, diagnostic laboratory, and imaging services obtained within 7 days of the index appointment

Multivariate analyses showed no significant effect of scheduled appointment length on repeat visits (OR = 0.983, CI: 0.873,1.106) or ED visits (OR = 0.856, CI: 0.700, 1.047) (Fig. 1). Indeed, the strongest risk factor for repeat visits and subsequent ED visits was a history of greater ED utilization in the 6 months prior to the index visit (see Additional File 4, Tables D1-D2). Shorter appointment lengths were associated with a lower likelihood of subsequent hospitalizations (OR = 0.689, CI: 0.504, 0.941), laboratory services (OR = 0.682, CI: 0.643, 0.724), and diagnostic imaging services (OR = 0.499, CI: 0.466, 0.534) compared to longer appointment lengths (Fig. 2). Female patients were more likely to have subsequent laboratory services compared to male patients (OR = 1.296, CI: 1.184, 1.419) (see Additional file 4, Tables D3-D7). No other measures were significantly associated with our outcomes of interest.

None of the upper confidence limits for repeat visits (UCL = 1.106), laboratory services (UCL = 0.724), and diagnostic imaging services (UCL = 0.534) exceeded the non-inferiority threshold of an OR of 1.2 (i.e., 20% higher likelihood) (Fig. 2). Similarly, the upper confidence limits for subsequent ED visits (UCL = 1.047) and hospitalizations (UCL = 0.941) fell below the non-inferiority threshold of an OR of 1.1 (i.e., 10% higher likelihood) (Fig. 1). These findings indicate that shorter scheduled appointments are non-inferior to longer scheduled appointments for all primary study outcomes.

## Discussion

Real-time evaluation of practice changes to assess for unanticipated and undesired outcomes is necessary to ensure that care delivery is safe, timely, effective, equitable, efficient and patient-centered [40]. Our study aimed to fill a critical knowledge gap by assessing whether 15-min primary care appointments represent a non-inferior option to traditional 30-min or longer appointments with respect to need for repeat primary care visits, ancillary diagnostic studies (laboratory and imaging tests), and ED visits and hospitalizations. We found that when propensity score matched on important patient and visit characteristics, patients seen for 15-min appointments did not incur greater healthcare utilization within seven days of the index visit, demonstrating that shorter appointments may provide a non-inferior option for scheduling when used for carefully selected patient populations.

Ideally, primary care appointments would allow for enough time to successfully complete all necessary clinical and ancillary tasks without shifting care to later appointments or generating unnecessary diagnostic testing or referrals. While the time required to complete all tasks associated with a comprehensive primary care appointment is increasing, healthcare organizations may be pressured to limit appointment durations to maximize access and reimbursement. Our findings suggest that for carefully selected patients with low-risk visit indications—the situations where these appointments were being used—shorter appointment lengths do not result in greater down-stream healthcare utilization. However, as only a small number of total primary care appointments in our study period were of shorter length and were focused on simpler chief complaints and lower risk patients, our findings should not be generalized to higher risk patients or chief complaints that were excluded from the comparisons during matching. Thus, changes to scheduling standards should be considered with caution to avoid potential oversaturation of shorter appointments within provider calendars, which could limit their ability to effectively manage patients scheduled on a given day.

Prior work in evaluating appointment lengths has found an association between increasing appointment lengths and improved quality of care indicators, better counseling or screening, higher patient satisfaction, and lower risk of malpractice suits, as longer appointments allow adequate time to perform comprehensive services [11, 20]. Similarly, other retrospective work found that shorter appointment lengths are associated with incomplete visit tasks and higher medication prescribing, serving as a surrogate for lower value care, albeit much of this work was completed outside of the United States [30, 32, 41, 42]. Our study built on these findings to offer reassurance that for select lower risk conditions and patients, shorter appointment lengths do not necessarily translate to greater total healthcare utilization secondary to incomplete or incorrect diagnostic evaluations. However, we did not consider whether longer appointments were more conducive to addressing health maintenance and preventive health needs; while this is not indicative of suboptimal care for the acute condition serving as the chief complaint, it nevertheless reflects missed opportunities to deliver care and improve long-term health outcomes.

To our knowledge, this is the first contemporary study to examine the impact of scheduled appointment lengths on subsequent healthcare utilization in the general primary care population in the United States. It was made uniquely possible by linking EHR data, which spans the outpatient and inpatient settings, to administrative scheduling data, which allowed us to examine healthcare utilization and outcomes for a diverse population of primary care patients. Our analyses are further enhanced by the use of administrative scheduling data, which includes the actual durations allotted to specific appointments, rather than CPT codes, which are imperfect surrogates of time spent on patient care and do not necessarily reflect the time allotted for the care that was performed. As part of a regionally dominant, integrated healthcare system we suspect there is very little leakage of down-stream care to other healthcare systems unable to be captured in our data. The five included clinics represented both urban and rural settings, patients were seen in teaching and non-teaching clinics, and a variety of practice styles and clinic level resources were available across the different sites, increasing the generalizability of our findings.

While informative, this study is subject to some limitations. Because this is an observational study using secondary data analysis approaches, we are limited to describing associations present in the data and cannot make causal inferences. To minimize the impact of this limitation, we utilized propensity score matching, which is a common approach to address underlying confounding and selection bias to estimate causal effects. However, because propensity score matching relies on observable data and administrative data alone cannot fully capture patient and care complexity and key social determinants of health, there may be residual bias in our study, resulting in some populations not being matched and included in our outcomes assessments. Thus, there may be important subgroups of patients, particularly those with multiple chronic conditions, patients with limited English proficiency who require interpreter services, patients with psychosocial barriers to health and healthcare, and those seen by trainee clinicians, for whom longer appointments may remain the superior option for scheduling. Additionally, care managed through email, phone calls, patient portals, and telemedicine is not represented in our study. Therefore, inferences of this study are only generalizable to face-to-face visits with the potential to be scheduled at a 15-min interval.

Further research in this area is needed to comprehensively understand how appointment scheduling approaches impact the clinical experience. While we showed non-inferiority of shorter appointments in this population as it relates to subsequent healthcare utilization, the association of appointment lengths with patient satisfaction, chronic disease outcomes, and measures of physician burnout are less clear [20]. Investigation into the value of using more patient and physician centered scheduling templates may represent another research area of opportunity.

## Conclusions

Understanding how primary care appointment lengths impact downstream care and utilization may be of significant value to clinicians, practice administrators, quality improvement professionals, payers, and health policy experts. This study investigated the association of scheduled appointment length on repeat visits and diagnostic testing services rendered within the 7 days following the appointment and demonstrated that under the specific circumstances being considered, shorter appointment lengths, in carefully selected patients and carefully selected conditions may be adequate to meet patient needs. These findings can be used to improve healthcare delivery models and triage processes to provide higher quality and more efficient care, while aiming to reduce low-value healthcare utilization.

## Supplementary Information

**Additional file 1.** Scheduling Template**. **Standardized scheduling template used by centralized scheduling staff members when assigning appointment lengths.

**Additional file 2.** Billing Codes Used to Define Outcomes of Interest**. **Billing code rules used to identify laboratory and diagnostic imaging services outcomes.

**Additional file 3.** Technical Appendix – Propensity Score Matching. A detailed summary of the methods used to carry out the propensity score matching approach.

**Additional file 4.** Full Regression Model Results**. **A full summary of regression model estimates for each of the outcomes of interest.

## Data Availability

The datasets generated and/or analyzed during the current study are not publicly available due to inclusion of protected health information but can be made available subsequent to de-identification upon reasonable request.

## Abbreviations

PCP: Primary care providers
EHR: Electronic health record
NP: Nurse practitioners
PA: Physician assistant
CIM: Community internal medicine
FM: Family medicine
CPT: Current procedural terminology
E&M: Evaluation and management
ED: Emergency department
CCSR: Clinical classification software refined
OR: Odds ratio
UCL: Upper confidence limit
