# Supplementary material for: Association between primary care appointment lengths and subsequent ambulatory reassessment, emergency department care, and hospitalization: a cohort study
Source: BMC Prim Care. 2022 Mar 6;23:39. doi: 10.1186/s12875-022-01644-8 (PMC8900401; doi:10.1186/s12875-022-01644-8)
Supplement: Supplementary file 1 — Additional file 1. Scheduling Template. Standardized scheduling template used by centralized scheduling staff members when assigning appointment lengths. [file 12875_2022_1644_MOESM1_ESM.docx]

**Appointment Scheduling Template: criteria used by centralized scheduling staff members to determine the appointment length to schedule**

|  | **Patient age 18-64 years** | **Patient age ≥65 years** |
| --- | --- | --- |
| **15 minutes** | Dermatologic concerns, eye concerns, ear concerns, gastrointestinal concerns, respiratory concerns (cough [not with Asthma or COPD], sinusitis, allergies), STD check, UTI | Dermatologic concerns, eye concerns, ear concerns, gastrointestinal concerns, respiratory concerns (sinusitis, allergies), STD check, UTI |
| **30 minutes** | Mental health, edema, ED or hospital follow-up, fatigue, fainting, dizziness, gynecological/obstetrical concerns, medication checks, obesity, multiple symptoms, Pain, Pre-Op, respiratory concerns (with Asthma/COPD/shortness of breath), Sleep, orthopedic | Same as 18-64 plus cough/upper respiratory infection with or without Asthma/COPD/shortness of breath |
| **45 minutes** | New patient to primary care, Interpreter required, Physical exam with 3 or more chronic conditions (18-64), All physical exams 65 or older | |
