# Supplementary material for: Association between primary care appointment lengths and subsequent ambulatory reassessment, emergency department care, and hospitalization: a cohort study
Source: BMC Prim Care. 2022 Mar 6;23:39. doi: 10.1186/s12875-022-01644-8 (PMC8900401; doi:10.1186/s12875-022-01644-8)
Supplement: Supplementary file 2 — Additional file 2. Billing Codes Used to Define Outcomes of Interest. Billing code rules used to identify laboratory and diagnostic imaging services outcomes. [file 12875_2022_1644_MOESM2_ESM.docx]

**Billing Codes Used to Define Outcomes of Interest**

| Outcome Category | Revenue Center Codes | CPT-4 Codes |
| --- | --- | --- |
| Diagnostic Laboratory | '0300', '0301', '0302', '0303', '0304', '0305', '0306', '0307', '0309', '0310', '0311', '0312', '0314', '0319', '0923', '0925', '0971' | N/A |
| Diagnostic Imaging | '0320', '0321', '0322', '0323', '0324', '0329', '0340', '0341',  '0350', '0351', '0352', '0359', '0400', '0401', '0402', '0403', '0404', '0409', '0460', '0469', '0470', '0471', '0482', '0483',  '0610', '0611', '0612', '0614', '0615', '0616', '0618', '0619',  '0730', '0731', '0732', '0739', '0740', '0749', '0750','0921', '0922', '0924', '0972', '0974', '0985', '0986' | N/A |
|  | '0929' | '93784', '93786', '93799', '93799' |
