# Supplementary material for: Association between primary care appointment lengths and subsequent ambulatory reassessment, emergency department care, and hospitalization: a cohort study
Source: BMC Prim Care. 2022 Mar 6;23:39. doi: 10.1186/s12875-022-01644-8 (PMC8900401; doi:10.1186/s12875-022-01644-8)
Supplement: Supplementary file 3 — Additional file 3. Technical Appendix – Propensity Score Matching. A detailed summary of the methods used to carry out the propensity score matching approach. [file 12875_2022_1644_MOESM3_ESM.docx]

**Technical Appendix – Propensity Score Matching**

We used a variety of factors to build a propensity score model with the purpose of addressing the anticipated selection bias in patients received 15 versus 30 minute or longer appointments. Propensity scores were estimated using a logistic regression model predicting whether the patient was likely to receive a shorter (15-minute) or longer (30 minute and longer) appointment length. Factors used in the propensity score model to predict appointment length selection included age, practice area in which the appointment was scheduled, provider type, the language spoken by the patient, the clinic site, the severity weighted Charlson comorbidity index, and disease indication (i.e., primary diagnosis for the appointment). We also included interaction terms between the provider and clinic site, patient age and disease indication, patient age and practice area, patient age and provider type, as well as between patient age and clinic site. These interaction terms helped account for varying compositions of patients and providers within certain practice areas or clinics and, thus, varying likelihood of receiving a shorter or longer appointment.

We used the logit of the propensity score to match patients receiving 15-minute appointments to a group of comparable patients receiving 30 minute or longer appointments. We checked for adequate overlap in distribution of the propensity score across groups prior to matching to ensure that quality pairs would be created. The overlap of the logit of the propensity scores is shown in Figure A1

**Figure A1: Distribution of the logit of the propensity score across exposure groups (15 min. versus ≥ 30 min.)**


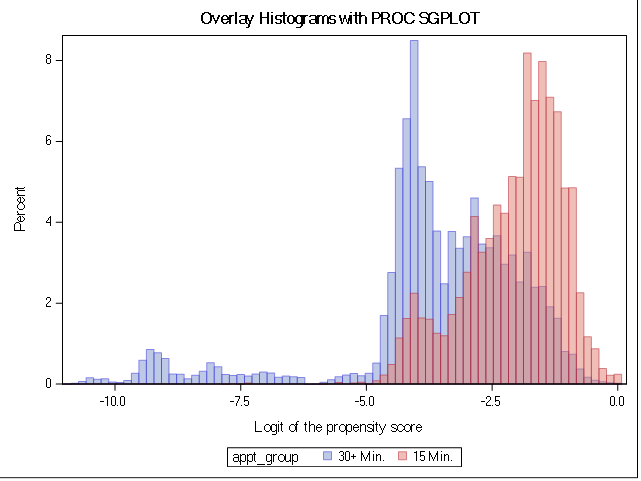


We performed a one-to-one match using a nearest neighbor method within a defined caliper (e.g., the maximum distance in which matches were allowed to be made). The caliper was defined using methods identified by Rosenbaum and Rubin.(1) In other words, we multiplied 0.2 times the square root of the pooled standard deviation of the logit of the propensity score. This value was used as our caliper (i.e., maximum allowable distance to identify a match) in our matching algorithm, which used the GREEDY algorithm where the smallest distance was defined as the weighted sum of the absolute differences between the case and control matching factors (i.e., the logit of the propensity score). We compared the standardized differences of the baseline covariates across the matched groups in the main body of our paper to verify that we had achieved adequate balance in the exposure groups.(2, 3) Unique ids were created to identify each case-control pairing to account for the matched nature of the data in all subsequent analyses (e.g., conditional logistic regression).

1. Rosenbaum PR, Rubin DB. Constructing a Control Group Using Multivariate Matched Sampling Methods That Incorporate the Propensity Score. The American Statistician. 1985;39:33-8.

2. Austin PC. An Introduction to Propensity Score Methods for Reducing the Effects of Confounding in Observational Studies. Multivariate Behav Res. 2011;46(3):399-424.

3. Austin PC. Balance diagnostics for comparing the distribution of baseline covariates between treatment groups in propensity-score matched samples. Stat Med. 2009;28(25):3083-107.
