# Supplementary material for: Association between primary care appointment lengths and subsequent ambulatory reassessment, emergency department care, and hospitalization: a cohort study
Source: BMC Prim Care. 2022 Mar 6;23:39. doi: 10.1186/s12875-022-01644-8 (PMC8900401; doi:10.1186/s12875-022-01644-8)
Supplement: Supplementary file 4 — Additional file 4. Full Regression Model Results. A full summary of regression model estimates for each of the outcomes of interest. [file 12875_2022_1644_MOESM4_ESM.docx]

**Full Regression Model Results**

**Table D1: Multivariate hierarchical logistic regression results assessing the effects of appointment length on repeat primary care visits**

|  |  | **Repeat Visits** | | |
| --- | --- | --- | --- | --- |
|  |  |  | **95% CI** | |
| **Variables** | | **Odds Ratio** | **Lower** | **Upper** |
| **Appointment Time** | |  |  |  |
|  | 15 | 0.983 | 0.873 | 1.106 |
|  | 30+ | Referent | Referent | Referent |
| **Ethnicity** | |  |  |  |
|  | Hispanic | 1.165 | 0.602 | 2.255 |
|  | Not Hispanic | Referent | Referent | Referent |
|  | Unknown | 0.944 | 0.544 | 1.636 |
| **Race** |  |  |  |  |
|  | African American | 0.480 | 0.205 | 1.124 |
|  | Asian | 0.982 | 0.530 | 1.819 |
|  | Other/Unknown | 1.180 | 0.728 | 1.912 |
|  | White | Referent | Referent | Referent |
| **Sex** |  |  |  |  |
|  | Female | 1.079 | 0.898 | 1.297 |
|  | Male | Referent | Referent | Referent |
| **Marital Status** | |  |  |  |
|  | Married | Referent | Referent | Referent |
|  | Single | 1.076 | 0.884 | 1.310 |
| **Number of Prior ED** | | 1.051 | 1.011 | 1.093 |
| **Number of Prior Hospitalizations** | | 1.035 | 0.877 | 1.222 |

**Table D2: Multivariate hierarchical logistic regression results assessing the effects of appointment length on subsequent emergency department visits**

|  |  | **ED Visits** | | |
| --- | --- | --- | --- | --- |
|  |  |  | **95% CI** | |
| **Variables** | | **Odds Ratio** | **Lower** | **Upper** |
| **Appointment Time** | |  |  |  |
|  | 15 | 0.856 | 0.700 | 1.047 |
|  | 30+ | Referent | Referent | Referent |
| **Ethnicity** | |  |  |  |
|  | Hispanic | 1.044 | 0.326 | 3.342 |
|  | Not Hispanic | Referent | Referent | Referent |
|  | Unknown | 0.343 | 0.093 | 1.258 |
| **Race** |  |  |  |  |
|  | African American | 2.498 | 0.735 | 8.494 |
|  | Asian | 1.139 | 0.434 | 2.988 |
|  | Other/Unknown | 0.744 | 0.292 | 1.895 |
|  | White | Referent | Referent | Referent |
| **Sex** |  |  |  |  |
|  | Female | 0.966 | 0.711 | 1.313 |
|  | Male | Referent | Referent | Referent |
| **Marital Status** | |  |  |  |
|  | Married | Referent | Referent | Referent |
|  | Single | 1.210 | 0.854 | 1.716 |
| **Number of Prior ED** | | 1.155 | 1.069 | 1.247 |
| **Number of Prior Hospitalizations** | | 0.903 | 0.669 | 1.217 |

**Table D3: Multivariate hierarchical logistic regression results assessing the effects of appointment length on subsequent hospitalizations**

|  |  | **Hospitalizations** | | |
| --- | --- | --- | --- | --- |
|  |  |  | **95% CI** | |
| **Variables** | | **Odds Ratio** | **Lower** | **Upper** |
| **Appointment Time** | |  |  |  |
|  | 15 | 0.689 | 0.504 | 0.941 |
|  | 30+ | Referent | Referent | Referent |
| **Ethnicity** | |  |  |  |
|  | Hispanic | 0.701 | 0.057 | 8.589 |
|  | Not Hispanic | Referent | Referent | Referent |
|  | Unknown | 0.285 | 0.055 | 1.479 |
| **Race** |  |  |  |  |
|  | African American | 1.137 | 0.153 | 8.462 |
|  | Asian | 0.495 | 0.077 | 3.175 |
|  | Other/Unknown | 0.330 | 0.031 | 3.490 |
|  | White | Referent | Referent | Referent |
| **Sex** |  |  |  |  |
|  | Female | 0.729 | 0.443 | 1.200 |
|  | Male | Referent | Referent | Referent |
| **Marital Status** | |  |  |  |
|  | Married | Referent | Referent | Referent |
|  | Single | 1.123 | 0.646 | 1.953 |
| **Number of Prior ED** | | 1.016 | 0.957 | 1.080 |
| **Number of Prior Hospitalizations** | | 1.378 | 0.986 | 1.924 |

**Table D4: Multivariate hierarchical logistic regression results assessing the effects of appointment length on subsequent diagnostic laboratory services**

|  |  | **Diagnostic Laboratory** | | |
| --- | --- | --- | --- | --- |
|  |  |  | **95% CI** | |
| **Variables** | | **Odds Ratio** | **Lower** | **Upper** |
| **Appointment Time** | |  |  |  |
|  | 15 | 0.682 | 0.643 | 0.724 |
|  | 30+ | Referent | Referent | Referent |
| **Ethnicity** | |  |  |  |
|  | Hispanic | 1.269 | 0.899 | 1.792 |
|  | Not Hispanic | Referent | Referent | Referent |
|  | Unknown | 0.954 | 0.738 | 1.234 |
| **Race** | |  |  |  |
|  | African American | 1.051 | 0.768 | 1.439 |
|  | Asian | 1.019 | 0.771 | 1.347 |
|  | Other/Unknown | 0.992 | 0.767 | 1.284 |
|  | White | Referent | Referent | Referent |
| **Sex** |  |  |  |  |
|  | Female | 1.296 | 1.184 | 1.419 |
|  | Male | Referent | Referent | Referent |
| **Marital Status** | |  |  |  |
|  | Married | Referent | Referent | Referent |
|  | Single | 1.010 | 0.915 | 1.116 |
| **Number of Prior ED** | | 1.015 | 0.992 | 1.038 |
| **Number of Prior Hospitalizations** | | 0.992 | 0.904 | 1.088 |

**Table D5: Multivariate hierarchical logistic regression results assessing the effects of appointment length on subsequent diagnostic laboratory services (excluding same day services)**

|  |  | **Diagnostic Laboratory (excluding same day services)** | | |
| --- | --- | --- | --- | --- |
|  |  |  | **95% CI** | |
| **Variables** | | **Odds Ratio** | **Lower** | **Upper** |
| **Appointment Time** | |  |  |  |
|  | 15 | 0.834 | 0.771 | 0.903 |
|  | 30+ | Referent | Referent | Referent |
| **Ethnicity** | |  |  |  |
|  | Hispanic | 1.459 | 0.897 | 2.370 |
|  | Not Hispanic | Referent | Referent | Referent |
|  | Unknown | 0.834 | 0.582 | 1.195 |
| **Race** | |  |  |  |
|  | African American | 0.972 | 0.628 | 1.506 |
|  | Asian | 1.032 | 0.698 | 1.526 |
|  | Other/Unknown | 0.889 | 0.619 | 1.276 |
|  | White | Referent | Referent | Referent |
| **Sex** |  |  |  |  |
|  | Female | 0.949 | 0.841 | 1.071 |
|  | Male | Referent | Referent | Referent |
| **Marital Status** | |  |  |  |
|  | Married | Referent | Referent | Referent |
|  | Single | 0.990 | 0.867 | 1.131 |
| **Number of Prior ED** | | 1.045 | 1.016 | 1.075 |
| **Number of Prior Hospitalizations** | | 1.092 | 0.979 | 1.218 |

**Table D6: Multivariate hierarchical logistic regression results assessing the effects of appointment length on subsequent diagnostic imaging services**

|  |  | **Diagnostic Imaging** | | |
| --- | --- | --- | --- | --- |
|  |  |  | **95% CI** | |
| **Variables** | | **Odds Ratio** | **Lower** | **Upper** |
| **Appointment Time** | |  |  |  |
|  | 15 | 0.499 | 0.466 | 0.534 |
|  | 30+ | Referent | Referent | Referent |
| **Ethnicity** | |  |  |  |
|  | Hispanic | 1.522 | 0.981 | 2.359 |
|  | Not Hispanic | Referent | Referent | Referent |
|  | Unknown | 0.716 | 0.535 | 0.957 |
| **Race** | |  |  |  |
|  | African American | 0.859 | 0.581 | 1.270 |
|  | Asian | 0.781 | 0.557 | 1.095 |
|  | Other/Unknown | 0.793 | 0.587 | 1.072 |
|  | White | Referent | Referent | Referent |
| **Sex** |  |  |  |  |
|  | Female | 1.084 | 0.979 | 1.201 |
|  | Male | Referent | Referent | Referent |
| **Marital Status** | |  |  |  |
|  | Married | Referent | Referent | Referent |
|  | Single | 0.903 | 0.805 | 1.015 |
| **Number of Prior ED** | | 1.019 | 0.995 | 1.044 |
| **Number of Prior Hospitalizations** | | 0.974 | 0.880 | 1.078 |

**Table D7: Multivariate hierarchical logistic regression results assessing the effects of appointment length on subsequent diagnostic imaging services (excluding same day services)**

|  |  | **Diagnostic Imaging (excluding same day services)** | | |
| --- | --- | --- | --- | --- |
|  |  |  | **95% CI** |  |
| **Variables** | | **Odds Ratio** | **Lower** | **Upper** |
| **Appointment Time** | |  |  |  |
|  | 15 | 0.563 | 0.518 | 0.612 |
|  | 30+ | Referent | Referent | Referent |
| **Ethnicity** | |  |  |  |
|  | Hispanic | 1.394 | 0.819 | 2.374 |
|  | Not Hispanic | Referent | Referent | Referent |
|  | Unknown | 0.690 | 0.477 | 0.999 |
| **Race** | |  |  |  |
|  | African American | 0.823 | 0.487 | 1.392 |
|  | Asian | 0.707 | 0.475 | 1.053 |
|  | Other/Unknown | 0.740 | 0.504 | 1.088 |
|  | White | Referent | Referent | Referent |
| **Sex** |  |  |  |  |
|  | Female | 1.139 | 1.004 | 1.291 |
|  | Male | Referent | Referent | Referent |
| **Marital Status** | |  |  |  |
|  | Married | Referent | Referent | Referent |
|  | Single | 0.867 | 0.753 | 0.998 |
| **Number of Prior ED** | | 1.032 | 1.003 | 1.062 |
| **Number of Prior Hospitalizations** | | 0.999 | 0.889 | 1.123 |
